# Prospects of herbivore egg‐killing plant defenses for sustainable crop protection

**DOI:** 10.1002/ece3.2365

**Published:** 2016-09-07

**Authors:** Nina E. Fatouros, Antonino Cusumano, Etienne G.J. Danchin, Stefano Colazza

**Affiliations:** ^1^ Biosystematics Group Wageningen University Droevendaalsesteeg 1 6700 AP Wageningen The Netherlands; ^2^ Laboratory of Entomology Wageningen University Droevendaalsesteeg 1 6708 PB Wageningen The Netherlands; ^3^ INRA CNRS, UMR 1355‐7254 Institut Sophia Agrobiotech University of Nice Sophia Antipolis 06900 Sophia Antipolis France; ^4^ Department of Agricultural and Forest Sciences University of Palermo Viale delle Scienze edificio 5 90128 Palermo Italy

**Keywords:** Egg deposition, egg parasitoids, hypersensitive response, oviposition‐induced plant volatiles, phylogeny

## Abstract

Due to a growing demand of food production worldwide, new strategies are suggested to allow for sustainable production of food with minimal effects on natural resources. A promising alternative to the application of chemical pesticides is the implementation of crops resistant to insect pests. Plants produce compounds that are harmful to a wide range of attackers, including insect pests; thus, exploitation of their natural defense system can be the key for the development of pest‐resistant crops. Interestingly, some plants possess a unique first line of defense that eliminates the enemy before it becomes destructive: egg‐killing. Insect eggs can trigger (1) direct defenses, mostly including plant cell tissue growth or cell death that lead to eggs desiccating, being crushed or falling off the plant or (2) indirect defenses, plant chemical cues recruiting natural enemies that kill the egg or hatching larvae (parasitoids). The consequences of plant responses to eggs are that insect larvae do not hatch or that they are impeded in development, and damage to the plant is reduced. Here, we provide an overview on the ubiquity and evolutionary history of egg‐killing traits within the plant kingdom including crops. Up to now, little is known on the mechanisms and on the genetic basis of egg‐killing traits. Making use of egg‐killing defense traits in crops is a promising new way to sustainably reduce losses of crop yield. We provide suggestions for new breeding strategies to grow egg‐killing crops and improve biological control.

## Introduction

A growing demand of an increasing world population, estimated to reach 9 billion people in 2050, requires a drastic increase of food production (Godfray et al. 2010; Foley et al. 2011). Crop losses caused by phytopathogens and insects account for 25–40% of the annual worldwide production (Beddington 2010; Popp et al. 2013; Sobhy et al. 2014). Pest outbreaks are largely due to climate change, vast monocultures, and insect adaptations to pesticides and crop resistance (Bebber et al. 2013; Balmer et al. 2014; Guedes et al. 2016). Since decades, synthetic pesticides are the most influential pest management tool. But pesticide use is highly controversial as they are toxicants that contaminate the environment and adversely affect living species (Guedes et al. 2016). Thus, it is imperative to find strategies to increase yields with preferably minimal impact on natural ecosystems, including a reduction in use of chemical pesticides. Biological control of insect herbivore attackers by natural enemies (van Lenteren 2012; Colazza et al. 2014) and exploitation of the genetic variation in resistance traits among wild relatives are two promising and sustainable ways to reduce pest damages (Broekgaarden et al. 2011; Palmgren et al. 2015). However, such pest management strategies often allow the pest to continue feeding; they begin to work only when damage has already occurred. Moreover, due to plant domestication, crop defense mechanisms are often lowered in favor of high‐yield traits, and plants become more susceptible than their wild ancestors (Palmgren et al. 2015).

The existing literature on plant resistance traits against insects is highly biased, almost exclusively focusing on sublethal traits that slower the growth of feeding herbivores by traits such as toxic or antidigestive compounds, leaf toughness or trichomes (Schoonhoven et al. 2005; Agrawal 2011; Voelckel and Jander 2014) or attraction of larval parasitoids by herbivore‐induced plant volatiles (HIPVs) (Dicke and Baldwin 2010). Furthermore, many larvae of herbivores are mobile and can easily escape such defenses by moving to a neighboring plant.

**Figure 1:**
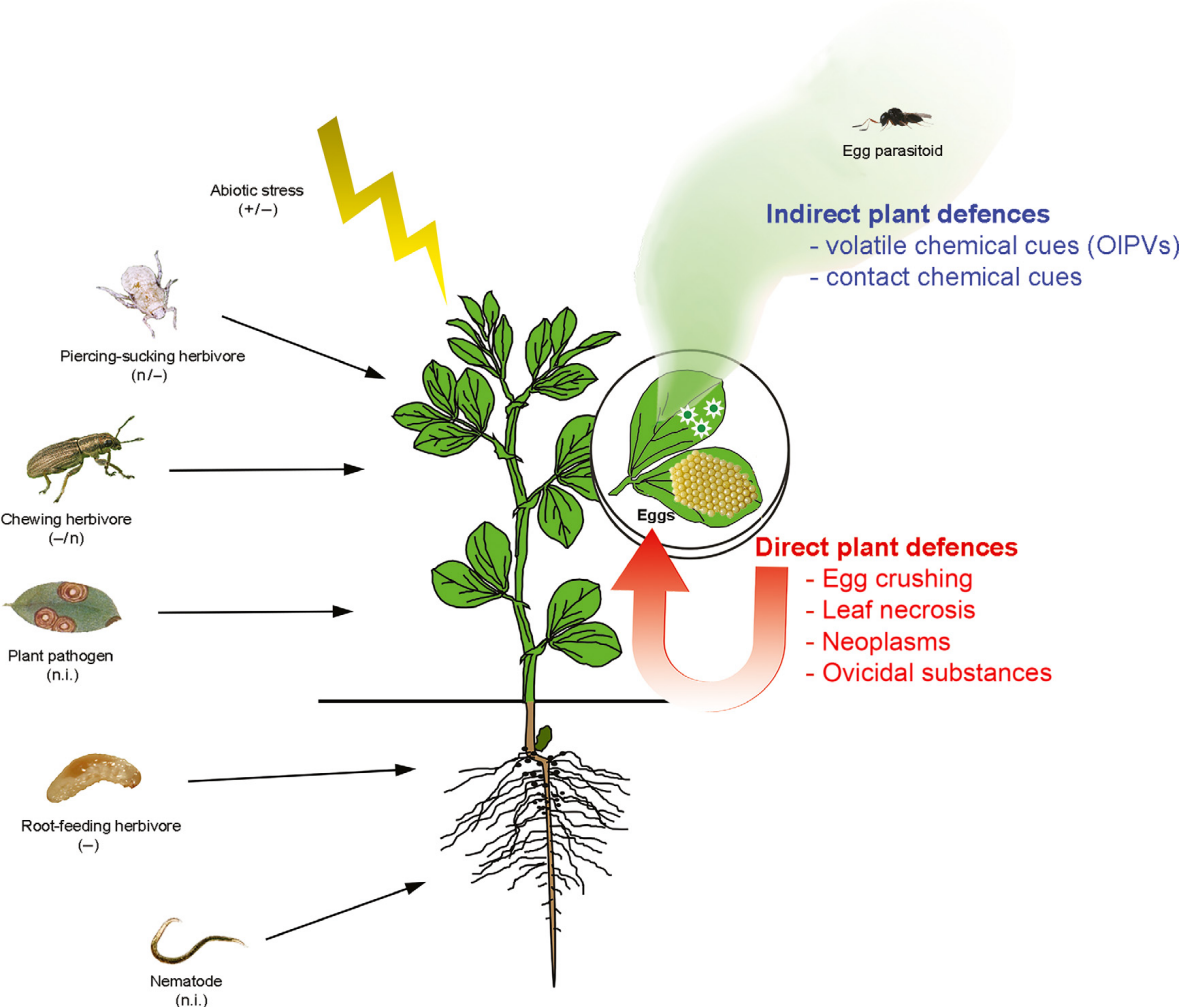
Known (a)biotic stressors affecting oviposition‐induced indirect defenses, that is, volatile chemical cues = oviposition‐induced plant volatiles and contact chemical cues recruiting egg parasitoids. Direct defenses against insect eggs have not been tested in a multiple stressor scenario. “+”, positive effect; “−”, negative affect; “n”, neutral effect; “n.i.”, not investigated.

In contrast, most lethal traits target immobile, nonfeeding stages, like eggs deposited on plants. Thus far, little attention has been paid to insect egg‐killing traits of plants that act before the pest causes damage. Such a plant defense strategy has been labeled “early herbivore alert” (Hilker and Meiners 2006). Plants that are able to respond to insect egg deposition can either directly defend themselves by targeting the eggs or defend indirectly by recruiting egg parasitoid wasps (Fig. 1). An increasing number of studies show that plants defend themselves against eggs of insects deposited on different plant tissues (Hilker and Fatouros 2015).

So far, direct egg‐killing defense traits have been described in diverse plant species, including crops, that either physiologically kill the eggs (Seino et al. 1996) or respond with plant cell death (Shapiro and De Vay 1987; Fatouros et al. 2012, 2014) or cell growth (Desurmont and Weston 2011; Petzold‐Maxwell et al. 2011) causing eggs to desiccate/drop off or being crushed, respectively. Some plants respond to herbivore egg deposition by releasing chemicals that recruit natural enemies such as egg parasitoids, that upon locating the herbivore host eggs, inject their own eggs and kill the host embryo to feed their own offspring (Fatouros et al. 2008; Colazza et al. 2010). However, under multiple herbivore scenarios, such plant stimuli can change and sometimes disrupt egg parasitoid recruitment (Moujahed et al. 2014; Cusumano et al. 2015) (Fig. 1).

**Figure 2:**
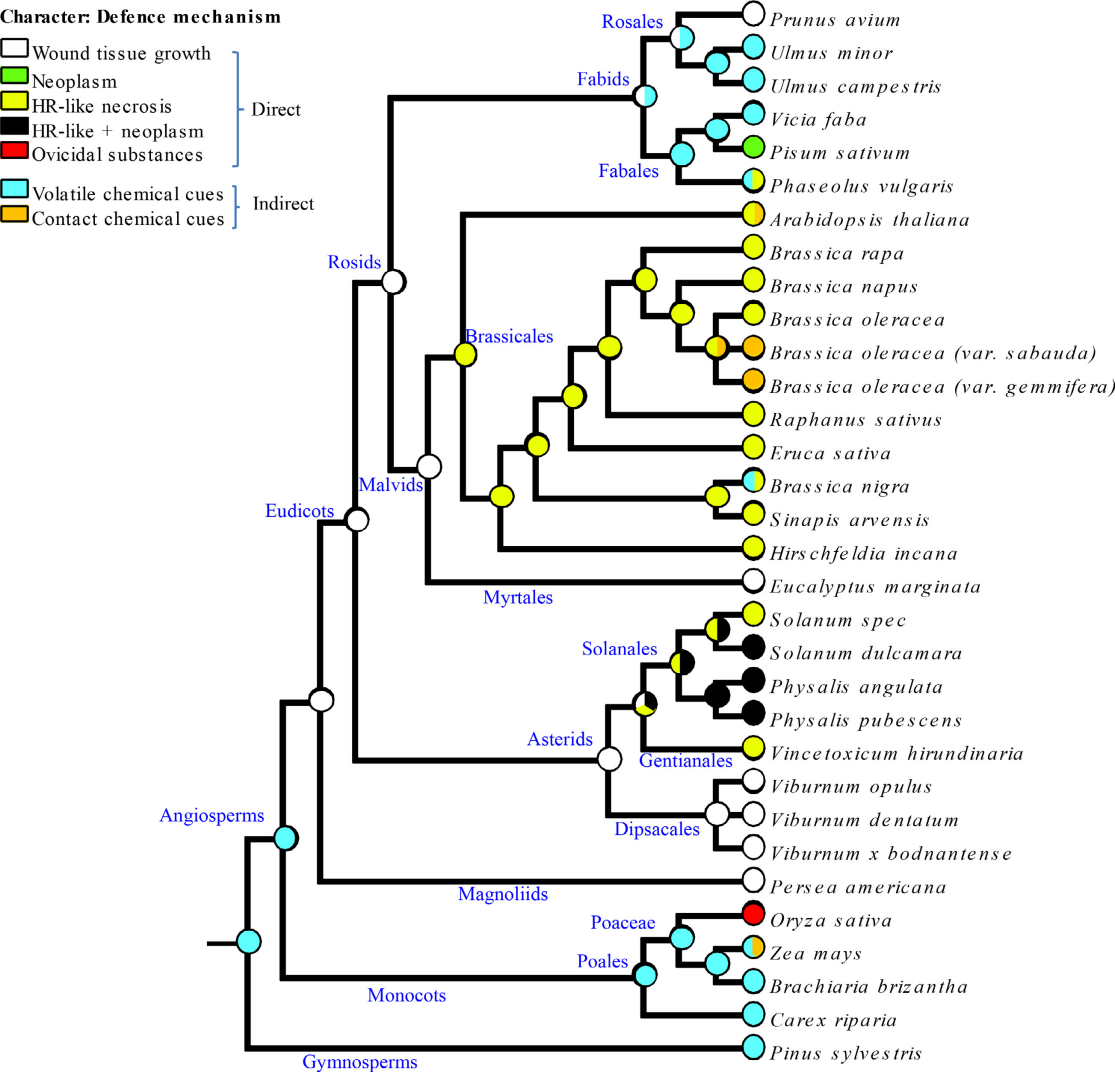
Reconstruction of the phylogeny of oviposition‐induced defense traits in 32 plants. The seven possible egg‐killing defense traits (five direct and two indirect) are represented at leaves and nodes of the tree according to the indicated color code. Whenever two different traits were observed within a same species, two colors are represented at a given leaf. More than one color at any ancestral node means that several ancestral states were equally parsimonious. Names of clades are indicated in blue along the branches.

Up to now, about 30 plant species belonging to different plant orders are known to express egg‐killing traits (Table 1). We mapped the distribution of these egg‐killing traits on a phylogeny of these species to get an understanding how ancient, widespread, and ubiquitous these traits are within the plant kingdom (Fig. 2). Exploiting egg‐killing defense traits should be promising to reduce losses of diverse types of crops in future. While recent reviews by Reymond (2013) and Hilker and Fatouros (2015, 2016) thoroughly discuss the mechanisms of egg‐killing traits, in this review article, we discuss the latest developments in research on egg‐killing traits including the research needed to create breeding strategies for egg‐killing insect‐resistant crops and improvements to biological control.

**Table 1:** Overview on plant species that employ different types of egg‐killing defenses induced by different herbivore species

Plant species	Defense type	Defense mechanism	Herbivore attacker	Reference
Angiosperms				
Family Adoxaceae				
*Viburnum opulus*	Direct	Wound tissue growth	*Pyrrhalta viburni*	Desurmont and Weston (2011)
*Viburnum dentatum*	Direct	Wound tissue growth	*P. viburni*	Desurmont and Weston (2011)
*Viburnum x bodnantense*	Direct	Wound tissue growth	*P. viburni*	Desurmont and Weston (2011)
Family Apocynaceae				
*Vincetoxicum hirundinaria*	Direct	HR‐like necrosis	*Abrostola asclepiadis*	Kalske et al. (2014)
Family Solanaceae				
*Physalis angulata*	Direct	HR‐like necrosis + neoplasm	*Heliothis subflexa*	Petzold‐Maxwell et al. (2011)
*Physalis pubescens*	Direct	HR‐like necrosis + neoplasm	*H. subflexa*	Petzold‐Maxwell et al. (2011)
*Solanum spec*. (cultivar)	Direct	HR‐like necrosis	*Leptinotarsa decemlineata*	Balbyshev and Lorenzen (1997)
*Solanum dulcamara*	Direct	HR‐like necrosis + neoplasm	*Different moth species*	A. Steppuhn, pers. comm.
Family Brassicaceae				
*Arabidopsis thaliana (Col‐0)*	Indirect	Contact chemical cues	*P. brassicae*	Blenn et al. (2012)
*Brassica napus* (cultivar)	Direct	HR‐like necrosis	*P. brassicae*	J.J.A. van Loon, pers. comm.
*Brassica nigra*	Direct	HR‐like necrosis	*P. rapae, P. brassicae, P. napi*	Shapiro and De Vay (1987); Fatouros et al. (2012, 2014)
	Indirect	Volatile chemical cues	*P. rapae, P. brassicae*	Fatouros et al. (2012, 2014); Cusumano et al. (2015)
*Brassica oleracea*	Direct	HR‐like necrosis	*P. brassicae*	Pashalidou et al. (2015a)
*Brassica oleracea var. sabauda* (cultivar)	Indirect	Contact chemical cues	*Murgantia histrionica*	Conti et al. (2010)
*Brassica oleracea var. gemmifera* (cultivar)	Indirect	Contact chemical cues	*P. rapae, P. brassicae*	Fatouros et al. (2005, 2008, 2009)
*Brassica rapa* (cultivar)	Direct	HR‐like necrosis	*P. rapae, P. brassicae*	Fatouros, unpubl. data
*Hirschfeldia incana*	Direct	HR‐like necrosis	*P. rapae, P. brassicae*	Fatouros, unpubl. data
*Raphanus sativus* (cultivar)	Direct	HR‐like necrosis	*P. rapae, P. brassicae*	Fatouros, unpubl. data
*Eruca sativa* (cultivar)	Direct	HR‐like necrosis	*P. brassicae*	Bruessow and Reymond (2007)
*Sinapis arvensis*	Direct	HR‐like necrosis	*P. rapae, P. brassicae*	Pashalidou et al. (2015a); Fatouros, unpubl. data
Family Fabaceae				
*Phaseolus vulgaris* (cultivar)	Direct	HR‐like necrosis	*Apion godmani*	Garza et al. (2001)
	Indirect	Volatile chemical cues	*Nezara virdidula*	Colazza et al. (2004a,b)
*Pisum sativum* (cultivar)	Direct	Neoplasm	*Callosobruchus maculatus, Bruchus pisorum*	Doss et al. (1995, 2000)
*Vicia fabia* (cultivar)	Indirect	Volatile chemical cues	*N. virdidula*	Colazza et al. (2004a,b)
Family Myrtaceae				
*Eucalyptus marginata*	Direct	Wound tissue growth	*Perthida glyphopa*	Mazanec (1985)
Family Ulmaceae				
*Ulmus minor*	Indirect	Volatile chemical cues	*Xanthogaleruca luteola*	Meiners and Hilker (2000)
*Ulmus campestris*	Indirect	Volatile chemical cues	*X. luteola*	Meiners and Hilker (1997)
Family Rosaceae				
*Prunus serotina*	Direct	Wound tissue growth	*Magicicada spp*.	Karban (1983)
Family Lauraceae				
*Persea americana* (cultivar)	Direct	Wound tissue growth	*Anastrepha spec*.	Aluja et al. (2004)
Family Poaceae				
*Brachiaria brizantha*	Indirect	Volatile chemical cues	*Chilo partellus*	Bruce et al. (2010)
*Oryza sativa* (cultivar)	Direct	Ovicidal substances	*Sogatella furcifera*	Seino and Suzuki (1997); Seino et al. (1996); Yang et al. (2013, 2014a,b)
*Zea mays* (cultivar)	Indirect	Volatile chemical cues	*C. partellus*	Tamiru et al. (2011, 2015)
*Zea mays* (cultivar)	Indirect	Contact chemical cues	*Sesamia nonagriodes*	Salerno et al. (2013)
Family Cyperaceae				
*Carex riparia*	Indirect	Volatile chemical cues	*Cicadella viridis*	Chiappini et al. (2012)
Gymnosperms				
Family Pinaceae				
*Pinus sylvestris*	Indirect	Volatile chemical cues	*Neodiprion sertifer, Diprion pini*	Hilker et al. (2002); Mumm et al. (2003)

## Direct: How Plants Can Directly Destroy Insect Eggs

Plants are capable of directly killing their enemies. Such lethal plant traits are mainly restricted to sessile herbivore stages that cannot escape the plant defense response, like eggs.

Some herbivore eggs induce responses in plants that resemble a hypersensitive response (HR), which is defined as a rapid cell death usually activated by pathogens resulting in necrosis restricting the pathogens to the inoculated regions (Lam et al. 2001). An HR‐like necrosis induced by herbivore insect eggs was first described in the wild crucifer *Brassica nigra*, a wild relative of cabbage crops, on which eggs of the small cabbage white butterfly/imported cabbage worm (*Pieris rapae*) were observed to desiccate and/or drop off the plants (Shapiro and De Vay 1987). Since then, HR‐like necrosis has been also observed in crop plants, induced by coleopteran pests like the bean‐pod weevil, *Apion godmani*, which often causes heavy losses in crops of common bean (*Phaseolus vulgaris*) (Garza et al. 2001), or the Colorado potato beetle, *Leptinotarsa decemlineata* on a hybrid potato variety (*Solanum* spec.) (Balbyshev and Lorenzen 1997). We show that egg deposition by the large cabbage white butterfly, *Pieris brassicae,* induces HR on different brassicaceous plants, including crop plants like the oilseeds *B. napus, B. rapa* or the radish *Raphanus sativus* (Fatouros et al. 2012; Pashalidou et al. 2015a; N.E. Fatouros, unpubl. data). To date, it is not exactly known what causes desiccation of egg by the plants. The most likely scenario is that, due to cell apoptosis underneath the egg, humidity drops and water is drawn out of the egg, which eventually leads to the egg shrinking (Shapiro and De Vay 1987; Clark and Faeth 1998).

Neoplasm formation in combination with HR‐like necrosis was also shown as egg‐killing responses in several solanaceous species: a callus grows below the eggs, dies, and falls of the plant and with it the insect egg. Oviposition of different moth species was shown to induce such responses in two ground‐cherry species (*Physalis spp*.) (Petzold‐Maxwell et al. 2011), and the bittersweet *Solanum dulcamara* (D. Geuss & A. Steppuhn, pers. comm.). In *Viburnum* shrubs (Adoxaceae), twigs produce wound tissue in response to eggs of the Viburnum leaf beetle (*Pyrrhalta viburni*) laid into cavities, leading to beetle eggs being crushed inside the cavity (Desurmont and Weston 2011). Further wound tissue growth responses are known in eucalyptus (*Eucalyptus marginata*) (Mazanec 1985), black cherries (*Prunus serotina*) (Karban 1983), and avocado (*Persea americana*) (Aluja et al. 2004) in response to egg deposition of leaf miner, cicada, or tephritid fly pests, respectively (Table 1).

## Indirect: Volatile and Contact Chemical Cues Recruiting Parasitoids

Plants respond to egg deposition of herbivore insects by recruiting egg and larval parasitoids (Hilker and Fatouros 2015). From a pest control perspective, the recruitment of egg parasitoids plays a key role as the herbivore is killed before plant damage occurs (Colazza et al. 2014). Because of this, egg parasitoids are massively produced worldwide as biological control agents, although their efficiency in agro‐ecosystems is not always satisfactory in terms of pest population suppression (van Lenteren 2012). Increasing egg parasitoid efficiency could be achieved *via* manipulation of egg‐induced plant infochemicals that enhance egg parasitoid foraging abilities, but this promising strategy has not yet been implemented. Egg‐induced plant defenses guide egg parasitoids toward the plant infested with herbivore eggs either by volatile attractants from a distance or by contact chemical cues at short range (Hilker and Meiners 2006; Fatouros et al. 2008; Colazza et al. 2010).

Volatile chemicals released by plants after egg deposition are called oviposition‐induced plant volatiles (OIPVs). OIPVs often consist of complex mixtures of volatiles including green leaf volatiles, terpenoids, and isothiocyanates (Hilker and Fatouros 2015). Egg‐induced changes in the volatile blends usually result in quantitative alterations, which both enhance or reduce emission of specific compounds, depending on the case study (Hilker and Fatouros 2015). OIPVs are emitted by several plant species regardless of being annual or perennial, monocotyledons or dicotyledons, gymnosperms and angiosperms (Table 1) (Meiners and Hilker 2000; Hilker et al. 2002; Mumm et al. 2003; Colazza et al. 2004a,b; Tamiru et al. 2011; Fatouros et al. 2012). Depending on the herbivore species, OIPV emission occurs with or without plant wounding. For example, when lepidopteran species lay eggs on plants, no immediate leaf tissue damage is observed. Studies conducted on maize landraces (*Zea mays*) and black mustard (*B. nigra*) showed that egg deposition by lepidopteran pests resulted in the emission of OIPVs that attract polyphagous *Trichogramma* egg parasitoids as well as larval parasitoids that eventually kill the caterpillars (Tamiru et al. 2011; Fatouros et al. 2012, 2014; Cusumano et al. 2015; Ponzio et al. 2016). However, beetles and sawflies damage the plant by feeding prior to oviposition and/or ovipositional wounding. Oviposition in combination with wounding by elm leaf beetles on elm (*Ulmus minor* or *U. campestris*) and by pine sawflies on pine (*Pinus sylvestris*) also induces OIPVs attracting specialist egg parasitoids (*O. gallerucae* and *Closterocerus ruforum*, respectively) (Meiners and Hilker 2000; Hilker et al. 2002; Mumm et al. 2003; Beyaert et al. 2010). Other studies on the leguminous crops *Phaseolus vulgaris* and *Vicia faba* revealed that oviposition by the polyphagous stink bugs and leafhoppers, often in combination with wounding due to sucking‐feeding activity prior to oviposition, results in the release of OIPVs that attract oligophagous egg parasitoids (Colazza et al. 2004a,b; Chiappini et al. 2012).

Recently, the role of OIPVs has been investigated in plants suffering multiple stresses, particularly when an herbivore not attacked by the egg parasitoid (nonhost) is also feeding on the plant. A growing body of literature suggests that, under multiple herbivore attack, the emission of OIPVs can be altered depending on several aspects of the nonhost herbivore attack such as insect feeding guild (Cusumano et al. 2015), plant organ attacked (Moujahed et al. 2014), herbivore density (Ponzio et al. 2016), and plant–insect coevolution (Cusumano et al. 2015) (Fig. 1). Consequently, depending on the interplay of the plant–insect interactions, indirect egg‐induced plant defenses could be disrupted or withstand nonhost herbivore interference. Even if the case studies are limited, it seems that feeding guild of the nonhost plays an important role. Wounding of a plant by chewers can interfere with the plant's response to eggs and thus, with attraction to OIPVs by egg parasitoids. For example, in *V. faba* crops, chewing by the nonhost beetle *Sitona lineatus* was sufficient to disrupt egg parasitoid (*Trissolcus basalis*) attraction toward *N. viridula* egg‐induced volatiles. Interestingly, regardless if nonhost beetle chewing damage was inflicted by larvae feeding on roots, or by adults feeding on leaves, the composition of the OIPV blend was significantly altered resulting in a decrease in attraction of the wasps (Moujahed et al. 2014). Moreover, under detrimental abiotic conditions, *V. faba* can improve indirect defenses against egg deposition, reducing the chances of further stress by larval feeding. Egg parasitoid attraction toward OIPVs was enhanced by severe water stress conditions, whereas mild water stress conditions have an opposite effect (Colazza et al. 2015).

In a wild brassicaceous plant (*B. nigra*), leaf chewing by caterpillars of native (*P. brassicae*) and invasive alien herbivores (*Spodoptera exigua*) disrupt *Trichogramma* species attraction toward *P. brassicae* egg‐induced volatiles (Cusumano et al. 2015). On the contrary, attack by phloem‐feeding insects, such as aphids, appears to have minor interference effects in egg‐induced indirect plant defenses (Cusumano et al. 2015; Ponzio et al. 2016). Nonetheless, aphids can still disrupt the attraction of egg parasitoids when they are present in high numbers on the plant (A. Cusumano, unpubl. data), or when they attack the same leaf bearing the *Pieris* eggs (Ponzio et al. 2016), suggesting a density‐dependent or local interference effect.

In addition to OIPVs, plants can respond to herbivore oviposition by changing chemical cues on the leaf surface, which are perceived by egg parasitoids after landing (Fatouros et al. 2005, 2009; Conti et al. 2010; Pashalidou et al. 2010; Blenn et al. 2012). This strategy appears quite effective as plants can inform natural enemies through volatile and/or contact chemical cues, thus increasing the probability that herbivore eggs are found and destroyed by egg parasitoids. Substrate‐borne chemical cues (Colazza et al. 2014) have been demonstrated in crops (maize, savoy cabbage) and wild brassicaceous plants resulting in alteration of the leaf chemistry composition. To date, only Blenn et al. (2012) investigated the nature of such chemical changes, showing that quantitative differences in epicuticular wax composition in *Arabidopsis thaliana* retained *Trichogramma* wasps to egg‐infested leaves. In particular, leaves induced by cabbage white butterfly eggs had higher quantities of tetratriacontanoic acid and lower quantities of tetracosanoic acid compared to clean control leaves.

## Effects of Egg‐Induced Resistances on Subsequent Attackers

Besides directly affecting herbivore insect eggs, recent studies have demonstrated that “early herbivore alert” responses can also increase defense against feeding stages (Hilker and Fatouros 2015, 2016) or even pathogens (Hilfiker et al. 2014). Evidence is growing that priming of stress responses by environmental cues that indicate future stress is common in plants but also other organisms lacking a nervous system such as fungi or bacteria (Hilker et al. 2015). Herbivore insect eggs are a reliable indicator for larvae to hatch within a defined period of time. In several plants, priming by insect eggs has been shown to reduce fitness proxies such as larval and pupal weight (Pashalidou et al. 2013, 2015a, b) and/or survival (Beyaert et al. 2011; Geiselhardt et al. 2013; Austel et al. 2016; Bandoly et al. 2015; Bandoly et al. 2016) and even reproductive capacity (Austel et al. 2016). Besides reduced herbivore performance, priming by eggs also enhances volatile emissions and attraction of larval parasitoids that lead to higher parasitism rates and benefit plant fitness in terms of higher seed production (Pashalidou et al. 2015b,c).

Remarkably, egg deposition can activate similar responses that are also triggered by pathogens such as the accumulation of the plant hormone salicylic acid (SA) (Little et al. 2007). In *Arabidopsis thaliana*, oviposition by *P. brassicae* activates a systemic required resistance response (SAR), which inhibits the growth of *Pseudomonas syringae* strains (Hilfiker et al. 2014). Although so far not shown for crop plants, the fact that oviposition activates immunity against bacterial infections offers prospects that the concept of early herbivore alert could become highly attractive for breeding programs. Furthermore, the activation of SA‐related defense pathways by egg deposition could also harm insects that are affected by the same defense pathways, such as aphids. However, this potential oviposition‐mediated cross‐resistance effect has not been tested yet.

## Ubiquity of Egg‐Killing Traits

So far, little is known on the evolutionary history of the various plant defense traits against herbivore eggs. To explore this issue, we draw a dated phylogeny of the 32 plants listed in Table 1 according to a reference timetree of 639 taxa of seed plants (Zanne et al. 2014) as well as an online timescaled molecular phylogeny for 32,223 land plant species (http://www.onezoom.org/vascularplants_tank2013nature.htm). The dated phylogeny and associated defense traits, according to Table 1, were imported to the software Mesquite (Maddison and Maddison 2014) for ancestral state reconstruction. We reconstructed ancestral defense traits according to the trait distribution observed today in these 32 plants, including 15 crop plants/cultivars or landraces, using the maximum‐parsimony method (Table 1). The distribution of defense traits regarding a direct and indirect egg‐killing strategy and inference of ancestral defense traits at each node of the tree are displayed in Figure 2. We are aware that this performed phylogenetic analysis is limited by the knowledge currently available in the literature: for instance, not all traits have been tested in all listed plants. Nevertheless, most plants have been tested with different insect species that differ in their egg‐laying mode (e.g. with or without ovipositional wounding, egg deposition of single eggs or in clusters), which can affect the plants’ response (Hilker and Fatouros 2015). Thus, despite these limitations, we conducted the first tentative phylogenetic analysis of egg‐killing traits within the plant kingdom to reveal the ubiquity and evolutionary history of these defense processes. Such information is of high importance for both basic and applied ecology.

The most parsimonious reconstruction proposes that the defense trait at the most ancestral node of this evolutionary tree was an indirect one, and more particularly the emission of volatile chemical cues (OIPVs). The taxa with the most outgroup positions in this analysis (i.e., gymnosperms and monocots) displayed this indirect defense trait; thus, it is logical that the inferred most ancestral trait was attraction of egg parasitoids by “volatile chemical cues”. Including more gymnosperms in a similar analysis would allow confirming whether this trait is really the most likely ancestral one among seed plants.

According to the distribution of defense traits, it appears as most parsimonious that the last common ancestor of eudicots had the direct defense trait “wound tissue growth”. In Brassicales, most of the species display an HR‐like necrosis (Shapiro and De Vay 1987; Fatouros et al. 2012, 2014, 2015; Pashalidou et al. 2015a) and this same trait was inferred as the most parsimonious in the ancestor of Brassicales.

It is interesting to note that the indirect defense trait egg parasitoid arrestment to “contact chemical cues” appears as a derived character in this evolutionary scenario. Indeed, this trait is represented in no ancestral node, and its sparse phylogenetic distribution rather suggests that it has evolved multiple times independently in different phyla. It is also interesting to point out that a lack of OIPV emission was mainly shown for different crop cultivars (*Brassica oleracea* or *Z. mays*), whereas their wild relatives or landraces emit OIPVs. The loss of OIPV emission could thus be a result of domestication. Indeed, in *Z. mays*, egg‐induced volatile emission is very rare in commercial hybrids but common in landraces (Tamiru et al. 2011, 2015).

## The Challenge of Enhanced Production of Parasitoid‐Attracting Cues in Crops

Since the discovery that plants respond to herbivore attack by releasing HIPVs that recruit natural enemies (Dicke and Sabelis 1988; Turlings et al. 1990), several researchers have suggested to exploit HIPVs to implement sustainable pest management programs. Manipulation of plant chemical cues is a promising strategy for biocontrol (Kaplan 2012) and can be obtained either by releasing synthetic HIPVs in agro‐ecosystems (James 2003) or by breeding plants for enhanced production of HIPVs after herbivore attack (Turlings and Ton 2006; Kappers et al. 2010). However, plant chemicals induced by herbivores have not been implemented so far in agro‐ecosystems despite several research efforts during the last decades. The only strategy in which plant chemical cues are opening a new realm for biological pest control is the “push and pull” system (Cook et al. 2006, 2007; Khan et al. 2010).

There are several reasons that have limited practical application of plant chemicals in agro‐ecosystems (Heil 2014). First of all, even if some supporting studies have been carried out (Schuman et al. 2012; Gols et al. 2015), there is still a debate about the fitness benefits of HIPVs for plants growing in both natural and agro‐ecosystems. Indeed, many parasitoids responding to HIPVs are koinobionts and thus do not immediately kill the herbivore. In this case, plants would suffer serious damage even when herbivores are successfully parasitized (Harvey et al. 2010; de Rijk et al. 2013; Balmer et al. 2014). In addition, HIPVs have been recently discovered to attract organisms belonging to the fourth trophic level (i.e., hyperparasitoids), which may counteract the plants’ benefit of recruiting natural enemies (Poelman et al. 2012). Another important aspect to be considered when designing biocontrol pest programs based on plant chemical manipulation is that HIPVs do not represent the resource used by natural enemies but the signal exploited to locate the herbivores. Consequently, natural enemies may learn to avoid plants overexpressing HIPVs when herbivores are not present on such plants with deleterious effect for biological pest control (Rodriguez‐Saona and Stelinski 2009; Kaplan 2012). However, to avoid such problem, the “attract‐and‐reward” approach has been recently proposed, in which natural enemies are first attracted by HIPVs and then rewarded with food resources (Simpson et al. 2011). There is growing interest in developing plants genetically engineered to release infochemicals for crop protection purposes (Ding et al. 2014; Bruce et al. 2015). However, a recent field study with wheat indicated that plants overexpressing HIPVs did not achieve the expected biological pest control, likely because the infochemical released continuously from uninfested plants may disrupt the attraction of natural enemies (Bruce et al. 2015).

Plant responses to insect oviposition have rarely been exploited for biological control programs despite the potential benefits of recruiting natural enemies before the herbivores feed on the crop. However, manipulation of direct and indirect plant defenses against herbivore egg deposition could be a timely and effective strategy. In fact, egg deposition constitutes a warning signal (early herbivore alert) that triggers egg‐killing responses in the plant of great potential for pest control (Hilker and Meiners 2006). Further studies should investigate whether OIPVs may have higher value for the plant than HIPVs considering that: (1) idiobiont parasitoids are likely to have a greater impact than koinobionts in terms of reducing plant damage inflicted by herbivore attacks (Fatouros et al. 2012); (2) OIPV emission can repel subsequent herbivore oviposition (Bruce et al. 2010; Fatouros et al. 2012); (3) koinobiont larval parasitoids are also attracted to OIPVs parasitizing those larvae that escaped from egg parasitism (Bruce et al. 2010; Fatouros et al. 2012; Pashalidou et al. 2015c).

## Exploiting Natural Variation in Egg‐Killing Resistances

Crop wild relatives, landraces, and old cultivars retain genetic variation for direct and indirect egg‐killing traits (Tamiru et al. 2011, 2015; Yang et al. 2014b). Such genetic variation in defenses possessed by wild ancestors could thus be used for producing crop plants resistant to pests opening new opportunities for biological control (Palmgren et al. 2015). In this perspective, wild crucifers represent an interesting system for “rewilding”. In fact, in the black mustard *B. nigra* but not in the cultivated *B. oleracea* var. *gemmifera*, a synergistic effect between direct and indirect egg‐induced plant defenses has been found. The synergistic use of two egg‐killing defense types was shown to lead to butterfly egg mortalities up to 80% in nature (Fatouros et al. 2014). This “double defense line” is a unique way to control insect pests and highly promising for crop protection.

Egg‐killing defenses differing between crop plants and their wild ancestors suggest that artificial selection may have caused the loss of defense traits (Chen et al. 2015; Tamiru et al. 2015). This hypothesis could be true especially when the selection process is aimed at increasing yield in crops subjected to pesticide treatments. There is a growing demand of sustainable food production worldwide. Breeding insect‐resistant crops may be a key alternative to chemical control (Palmgren et al. 2015). Plant defenses leading to immediate mortality of the pest before damage is inflicted, such as egg‐killing traits, are the most desired traits for breeders but mostly unexplored so far. Introgression of defense traits from wild species or landraces to cultivated plants with classical backcross methodology can be a powerful way to bring back lost defense traits again. When classical breeding may be difficult to achieve, genetic modification techniques could be also applied where current regulations allow (Zamir 2001). Regardless of the methodology, we believe that there is a high potential for pest control using egg‐killing plants.

## Identifying Molecular and Genetic Mechanisms for Resistance Breeding

A limiting aspect remains that the genetic and molecular mechanisms underlying egg‐induced defenses are far from being fully understood, despite the ample phenotypic evidence (Reymond 2013; Hilker and Fatouros 2015). Numerous resistance (*R*) genes involved in resistance against viruses, bacteria, fungi, oomycetes, nematodes, and sucking insects are characterized and efficiently used in crop improvement programs. So far, no *R* genes are known to be involved in the recognition of herbivore‐associated molecular pattern (HAMPs) from leaf‐chewing insects including caterpillars of generalist moths *Spodoptera* spp. or *Plutella xylostella,* which are destructive pests that also show increasing resistances to pesticides (Dhaliwal et al. 2010; Xia et al. 2013; Sharma 2014). Yet, two promising approaches are under investigation in order to unravel the genetic basis of a direct and indirect egg‐killing trait in graminaceous crops.

Yang et al. (2014b) are the first who studied the genetic and molecular basis of a direct resistance response of some japonica rice (*Oryza sativa*) varieties against egg deposition of a serious pest, the whitebacked planthopper (*Sogatella furcifera*). When eggs are laid into air spaces of leaf sheaths, they cause necrotic discolorations, or “watery lesions”, which contain an ovicidal substance, benzyl benzoate (Seino et al. 1996; Suzuki et al. 1996; Seino and Suzuki 1997; Yang et al. 2013, 2014a, b). First, they phenotyped the necrotic discoloration of egg‐infested leaf sheaths associated with egg mortality in double haploid rice lines derived from a resistant and susceptible cultivar. Then, they genotyped such lines by constructing a molecular linkage map revealing that 19 quantitative trait loci (QTLs) were associated with watery lesions and egg mortality. Such QTLs were located on 8 of the 12 chromosomes, and among them, *qWL6* was the major QTL. Further fine mapping in combination with a transcriptomic analysis defined a 122‐kb region on chromosome 6 containing four genes that were differentially regulated between the resistant and susceptible rice cultivar (Yang et al. 2014b). The information obtained from this study can be used as a starting point for breeding rice cultivars resistant to the whitebacked planthopper.

Tamiru et al. (2015) studied the phenotypic variation in volatile emissions of maize commercial hybrids and landraces induced by stemborer (*Chilo partellus*) oviposition attracting egg and larval parasitoids. In particular, in landraces, stemborer eggs induce increased emission of some terpenoids, including (*E*)‐4,8‐dimethyl‐1,3,7‐nonatriene (DMNT), a key compound for parasitoid attraction (Tamiru et al. 2011, 2015). Most commercial hybrids do not show an induction of parasitoid‐attracting compounds, suggesting a potential to breed‐in the indirect egg‐killing defense traits against stemborers expressed into maize lines showing high yield. To find genes that can be introgressed, Tamiru et al. (2015) are using genomewide association studies that map single‐nucleotide polymorphisms, gene markers that can be linked with the indirect egg‐killing defense trait.

Besides these two recent approaches, more studies are needed and we hope that future efforts will focus on the genetic aspects underlying lethal egg‐killing traits as such information could be the basis to develop a novel strategy for sustainable pest control.

## Conclusions/Outlook

In this review, we have highlighted the strategies adopted by plants to kill insect eggs, thus minimizing the damage inflicted as the herbivore is killed before the crop feeding stage. Such egg‐killing traits have been documented so far in about 30 plant species belonging to different plant orders, and this number is likely to increase rapidly as research in this area is still in its infancy. Our phylogenetic analysis supports the hypothesis that plant domestication negatively affected oviposition‐induced defense traits in brassicaceous and graminaceous crops, particularly the capacity of attracting parasitoids *via* OIPV emission seems lost in cultivated plants compared with wild relatives or landraces. However, our database is restricted to only a small subset of plant species, and expanding the knowledge on the evolutionary history of egg‐killing traits is necessary to fully understand the role played by artificial selection for high‐yield traits on plant defenses. Considering the advantages and the ubiquity of egg‐induced plant defenses, especially in wild species, we believe that egg‐killing traits have a strong potential to be implemented in pest control programs.

It is recommended to breed for inducible defenses rather than select for continuous expression of defenses in order to avoid costs when herbivores are not attacking the plant. Direct egg‐killing defenses are likely to be more attractive for plant breeders, who have traditionally focused on bitrophic interactions. Furthermore, as pest suppression is not dependent on the third trophic level, the results of implementing direct egg‐killing traits into crops are likely to be less variable and less context dependant. Breeding specifically for parasitoid‐attracting traits is more challenging, because the extra level of complexity represented by the actions of egg or larval parasitoids can increase the failure risks in controlling the pest population. Furthermore, as crops protected by these traits do not achieve the complete elimination of pests, farmers may be more interested in other alternatives for pest control. When possible, using an integrated approach in which crops are protected with both direct and indirect egg‐induced defenses is encouraged, as in the case of brassicaceous plants. Evidence accumulates that priming by egg deposition can enhance defenses against subsequent attack due to oviposition‐mediated cross‐resistance effects. Thus, crops with high resistances to eggs might also become better protected against feeding stages of pests or even pathogens. In a scenario in which multiple sustainable strategies are used, such crops equipped with egg‐killing traits can be supplemented with floral resources to maximize the pest control service provided by parasitoids using an attract‐and‐reward approach.

## Conflict of Interest

None declared.
